# Soil Microbial Life History Strategies Drive Microbial Carbon Use Efficiency Following Afforestation

**DOI:** 10.3390/microorganisms13122870

**Published:** 2025-12-17

**Authors:** Hongyan Cheng, Haoyuan Chong, Minshu Yuan, Chengjie Ren, Jun Wang, Fazhu Zhao

**Affiliations:** 1 Shaanxi Key Laboratory of Earth Surface System and Environmental Carrying Capacity, College of Urban and Environmental Science, Northwest University, Xi’an 710127, China; 2 Yangling Xinhua Ecology Technology Co., Ltd., Yangling 712100, China; 3 College of Agronomy, Northwest A&F University, Yangling 712100, China; 4 Shaanxi Key Laboratory for Carbon Neutral Technology, Northwest University, Xi’an 710127, China

**Keywords:** metagenomics, isotope, microbial C use efficiency, microbial life-history strategies, afforestation

## Abstract

Soil microbial carbon use efficiency (CUE) is the core of the soil carbon (C) cycle that captures a dual microbial control point between soil organic C (SOC) accumulation and loss. The interpretation of these patterns and drivers of microbial CUE after long-term afforestation remains, however, a major scientific challenge. In particular, there are major uncertainties about the role of microbial traits in driving CUE. Here, we compared sites along a 45-year afforestation chronosequence and combined the novel ^18^O-H_2_O tracer method with metagenomic analysis to quantify CUE and explore the mechanisms underlying microbe-mediated C dynamics. The results showed that soil microbial CUE significantly increased following afforestation and showed a positive relationship with SOC, which suggested that microbial CUE could promote C accumulation in afforested ecosystems. We further found the critical role of microbial traits in the regulation of CUE through altering microbial life history strategies: microbial CUE was positively and significantly correlated with resource acquisition (A) genes, but showed a negative and significant correlation with stress tolerance (S) strategy genes. These results suggested that soil microbes reduce investment in S strategies and shift to A and high yield (Y) strategies, thereby increasing CUE. This knowledge is important because it advances our understanding of the microbial physiological and evolutionary tradeoffs mediating soil C cycling in the context of human-induced land use change.

## 1. Introduction

Soil is the largest pool of terrestrial organic carbon (C) [1,2], which has a crucial ecosystem function in regulating the atmospheric C cycle [3,4]. Soil organic C (SOC) is coregulated by the fresh plant C inputs and microbial C metabolism, and sequestering C into soils as SOC can mitigate climate change [3,5]. Microbial C use efficiency (CUE) is defined as the ratio of soil C utilization for biomass production to total C uptake by microbes, an important parameter for regulating soil ‘C-microbes’ interactions [6]. The frameworks of microbial efficiency, matrix stabilization, and the microbial C pump suggest that a higher microbial CUE indicates a stronger ability to store soil C due to increased biomass synthesis and the availability of microbial residues for organic matter stability [3]. However, there are few measurements of microbial CUE during afforestation, lagging substantially behind those for macroorganisms, despite its crucial importance to terrestrial ecosystems and climate change. This is due to the scarcity of studies using a novel substrate-independent approach (^18^O-DNA synthesis during microbial growth) to accurately estimate microbial growth in the afforested context [7]. Therefore, it is necessary to evaluate microbial CUE changes in the afforested ecosystem based on the ^18^O method, which may be the key to improving the accuracy of predicting SOC storage dynamics.

Several studies have identified climate, plant input, and microbial properties as important drivers for microbial CUE [2,6,8]. Specifically, rising temperatures are expected to reduce microbial CUE by altering primary production and soil substrate availability, a shift that is typically linked to changes in soil microbial traits [2,8,9]. Microbes modulate energy allocation toward their metabolic processes or ecological strategies to determine microbial CUE [10,11]. Evidence of direct linkages between microbial CUE and microbial community diversity and composition has been reported [6,8]. However, a critical knowledge gap remains regarding the role of microbial physiological and evolutionary tradeoffs in regulating soil microbial CUE [12], particularly in the context of long-term afforestation. Microbial Y-A-S (high yield (Y), resource acquisition (A), and stress tolerance (S)) life history strategies represent sets of traits that tend to correlate due to physiological and evolutionary tradeoffs [13], with distinct strategies favored as environmental conditions change. This framework provides an opportunity to link microbial processes to ecosystem carbon fluxes [13,14]. Thus, elucidating the link between microbial CUE and microbial community traits from a life history strategy perspective is a key step toward advancing our understanding of how microbial traits regulate microbial CUE—further enhancing the predictive accuracy of soil C cycle models.

To address this gap, we collected soil samples from a 45-year age gradient of an afforestation chronosequence on the Loess Plateau. We aimed to investigate the relationship between ^18^O-derived CUE and microbial life history strategies (based on metagenomic assembly), thereby advancing our understanding of the regulatory mechanisms underlying microbial CUE. Previous studies have shown that soils with a higher C content have greater SOC stocks than soils with a lower C content due to microbial byproducts and necromass [5,15]. We hypothesized that an elevated microbial CUE may increase SOC along the afforestation chronosequence. In addition, microbial life history strategies encompass suites of traits that regulate microbial community functioning and have been incorporated into microbe-explicit models [13,14]. We further hypothesized that microbial life history strategies play a critical role in regulating microbial CUE. Thus, this study aimed to (i) characterize changes in microbial CUE and its relationship with SOC during afforestation; and (ii) identify the key drivers of microbial CUE—particularly the regulatory mechanisms by which microbial life history strategy traits govern microbial CUE.

## 2. Materials and Methods

### 2.1. Site Description and Sampling

Soil samples were collected from the Wuliwan catchment, located in Ansai County on the Loess Plateau, China (altitude 1061–1371 m above sea level, 36°51′ N; 109°20′ E), which is characterized by a typical temperate semi-arid climate. The mean annual temperature of the study site is 8.8 °C, and the mean precipitation is 505 mm. The forest type of the study site is *Robinia pseudoacacia*, which was planted to restore the degraded ecosystem [16]. The afforestation project was started in 1970 by the Institute of Soil and Water Conservation of the Chinese Academy of Science [17]. The soil in this study is Calcaric Cambisols (WRB).

Sampling was conducted during maximum vegetative cover and species richness (August 2019). The incremental borehole method was used to determine the age of the forest, and soil samples were collected from four different forest ages of *Robinia pseudoacacia* (14, 20, 30, and 45 years). Additionally, the active sloped farmlands growing millet using traditional cultivation techniques were used as references. At each forest site, three sampling plots were established (20 × 30 m, Table S1). For each plot, ten soil samples were collected by scraping in an ‘S’ shape at a depth of 0–10 cm, which were then mixed to provide one sample per replicate site. Subsequently, the soil samples were combined to form a composite sample. Fresh soil was sieved (2 mm) and homogenized, removing fine roots and other plant debris. Approximately 60 g dry weight soil (calculated from fresh soil) was used to analyze basic properties. The remaining soil from each composite sample (2–3 kg) was stored at 4 °C for the subsequent incubation experiments, and the other soil fraction was stored at −80 °C for DNA extraction.

### 2.2. Soil Properties Analysis

#### 2.2.1. Soil Physical and Chemical Analyses

Soil properties were analyzed using standard procedures [18]. Soil bulk density (BD) was determined using the weight and volume of a single core sample before and after drying. Soil moisture (SM) was assessed using the time it took for a single core sample to reach a predetermined mass in an oven set to 105 °C. SOC content was determined with the K_2_Cr_2_O_7_ oxidation method. Labile and stable C were determined through acid hydrolysis. Particulate organic matter fraction (POM) and mineral-associated organic matter fraction (MOM) were separated by density and size characteristics [19]. The density and size of POM were greater than 1.85 g cm^−3^ and 0.053 mm, respectively. MOM is the opposite. We separated POM and MOM from the soil by combining these characteristics. To be specific, 10 g of air-dried soil was dispersed in 100 mL of 5 g L^−1^ sodium hexametaphosphate solution by shaking for 18 h. The soil suspension was separated into MOM and POM by rinsing over a 53 μm sieve. These fractions were dried at 60 °C. Particulate organic C and mineral-associated organic C (MOC) were determined using the K_2_Cr_2_O_7_ oxidation method [18]. To further explore the mineral protection of SOC, we determined the content of OC (Ca-OC and Fe-OC) associated with Cation bridges and Fe- oxides, respectively. Ca-OC was extracted using the Na_2_SO_4_ solution [20]. Exchangeable Ca^2+^ (Ca_exe_) was extracted using 1 M ammonium acetate (pH 7.0) [21]. Fe-(hydr) oxides (Fe_d_, representing pedogenic Fe) and Fe-OC were determined using the citrate–bicarbonate–dithionite (CBD) method [22,23]. Ca_exe_ and Fe_d_ were determined with an inductively coupled plasma optical emission spectrometer (iCAP 6300, Thermo Scientific, Waltham, MA, USA). The detailed methodology is available in Appendix A and Appendix B.

#### 2.2.2. Soil Microbial Biomass and Enzyme Activity Analysis

Microbial biomass C (MBC) was measured using the fumigation–extraction technique [18]. First, fresh soil was fumigated with chloroform; second, soil MBC was extracted from fresh soil samples with 0.5 M K_2_SO_4_. The MBC concentration in the extracts was determined using a continuous flow analyzer (AA3; Bran+Luebbe GmbH, Norderstedt, Germany). *β*-1,4-glucosidase (BG, EC 3.2.1.21), β-1,4-D-cellobiohydrolases (CBH, EC3.2.1.91), peroxidases (PER, EC 1.11.1.7), and polyphenol oxidases (PO, EC 1.14.18.1) were considered to be C-degrading enzymes. These enzymes were determined from fresh soil samples using a modified method of standard fluorometric techniques [22,24]. In addition, the stoichiometric characteristics of soil total nutrients (SOC:TN, SOC:TP, and TN:TP) were calculated based on mass.

### 2.3. Microbial CUE

Microbial CUE was determined using the ^18^O-H_2_O method, which converts ^18^O from water to microbial DNA to avoid the effects of substrate addition. Before the isotope-labeled ^18^O experiment, the soil samples (15 g, dry weight) were placed in a 50 mL centrifuge tube and were adjusted to 60% water holding capacity by adding deionized water. All samples were then pre-incubated at 25 °C for 7 days to activate the microorganisms. After pre-incubation, two 500 mg soil samples from each plot were weighed into 2 mL chromatography vials. For one soil sample, ^18^O-H_2_O was added to reach 20 at% ^18^O enrichment in the final soil water and 60% of the water-holding capacity, while, for the other, the same amount of deionized water was added. The chromatography vials containing soil samples were placed in 20 mL headspace bottles, sealed with rubber plugs separately, and three empty headspace bottles were set as blanks. Afterward, CO_2_-free air was flushed for 3 min to make uniform initial gas conditions for the incubation systems. After incubation, about 10 mL of gas was extracted from the headspace bottle using a syringe and determined using a gas chromatograph (Agilent, Santa Clara, CA, USA, 7890B) for microbial respiration (R; μg C g^−1^ dry soil h^−1^). The DNA from the soil samples was extracted using the FastDNA SPIN Kit for Soil (MP Biomedicals). Then, the DNA extract was transferred to a silver cup and dried at 45 °C. Subsequently, the abundance of ^18^O and total O content were determined using an IRMS-TC/EA (Thermo Scientific) to calculate microbial growth (G; μg C g^−1^ dry soil h^−1^) as follows [8]:

(1) $${\mathrm{DNA}}_{\mathrm{produced}}=O_{\mathrm{total}}\times \frac{{\mathrm{at}\%}_{\mathrm{excess}}}{100}\times \frac{100}{{\mathrm{at}\%}_{\mathrm{lable}}}\times \frac{100}{31.21}$$

(2) $$F_{\mathrm{DNA}}=\frac{\mathrm{MBC}}{\mathrm{DNA}}$$

(3) $$\mathrm{Growth}=\frac{F_{\mathrm{DNA}}\times {\mathrm{DNA}}_{\mathrm{produced}}}{t}\;$$

(4) $$\mathrm{CUE}=\frac{\mathrm{Growth}}{\mathrm{Growth}+\mathrm{Respiration}}$$

where O_total_ is the total O content of the DNA extract, and at%_excess_ is the percentage of ^18^O excess (at%) in the labeled sample compared with the corresponding control. at%_label_ is the enrichment of ^18^O (at%) in the final soil solution. The constant 31.21 represents the proportional mass of oxygen content in DNA based on the average formula (C_39_H_44_O_24_N_15_P_4_). t represents the incubation time (h).

The biomass-specific respiration (*R_m_*), growth (*G_m_*), and microbial biomass turnover rates (MTR; year^−1^) were calculated as follows:

(5) $$R_{m}=\frac{\mathrm{Respiration}}{\mathrm{MBC}}$$

(6) $$G_{m}=\frac{\mathrm{Growth}}{\mathrm{MBC}}$$

(7) $$\mathrm{MTR}=\frac{{\mathrm{DNA}}_{\mathrm{produced}}\times 24\times 365}{{\mathrm{DNA}}_{\mathrm{content}}\times t}\;$$

where $\;{\mathrm{DNA}}_{\mathrm{content}}$ is the total DNA content of soil (μg g^−1^ dry soil).

### 2.4. DNA Extraction, Sequencing, and Data Processing

The FastDNA Spin kit (MP Biomedicals, Cleveland, OH, USA) was selected to extract DNA from 0.5 g fresh soil samples. The quality of the DNA extracts was examined using a spectrophotometer (NanoDrop 2000, Thermo Scientific, Waltham, MA, USA). Six replicates were performed for each soil sample to ensure sufficient DNA for shotgun metagenomic sequencing. The extracted microbial DNA was constructed into an Illumina TruSeq Nano DNA LT library with an Agilent metagenomic shotgun sequencing library, having an insertion size of 400 bp. We constructed metagenomic shotgun sequencing libraries from the extracted microbial DNA with a 500 bp insertion size using the Illumina TruSeq Nano DNA LT library preparation kit. The sequences can be found on the National Center for Biotechnology Information (NCBI), with the accession number SRP319300.

### 2.5. Metagenome Assembly and Life History Strategy

Raw sequencing reads were filtered to obtain high-quality reads for analysis [25]. Particularly, the sequencing adapter sequences were removed using Cutadapt (v4.x), and low-quality reads were trimmed using a sliding-window algorithm in fastp [26]. We used the megahit software (version 1.2.9) [27] to assemble the mixed sequences of all samples to obtain larger contigs and Scaffolds databases. To obtain the lowest common ancestor classification, non-redundant contigs were compared with the NCBI nucleotide database using BLASTN + 2.16.0, and contigs belonging to Viridiplantae or Metazoa were deleted. The contigs that were higher than 200 bp were annotated using MetaGeneMark [28] and MetaEuk [29] to predict genes.

Microbial Y life history strategies are characterized by microbial CUE [13]. A and S life history strategies are from the KEGG database. Based on the published literature, the functional genes of various C complexes, ranging from labile to stable C degradation, were defined as A life history strategies [21,30,31]. In general, monosaccharides, disaccharides, polysaccharides, hemicellulose, cellulose, and amino sugars were categorized as having labile C composition, while lipids, chitin, and lignin were categorized as having stable C composition. The number of sequences contained in different biological functions was counted. These marker genes for S life history strategies were manually inspected based on previous literature reports [32] and public databases such as KEGG and UniProt.

### 2.6. Statistical Analyses

Before data analysis, we checked the normality of the data and performed logarithmic transformations if necessary. We used one-way analysis of variance (ANOVA, SPSS 26 Inc., Chicago, IL, USA) to assess the change in microbial metabolic properties (CUE, MTR, growth (base soil or MBC), and respiration (base soil or MBC)). To investigate the relationship between microbial CUE and four groups of potential factors, such as A strategy genes, S strategy genes, soil enzymes, and C substrate, we introduced partial correlation analysis, which can evaluate numerous inter-correlated variables [33,34]. During the construction of the partial correlation analysis, principal component analysis (PCA) was performed on the factors within each group to reduce collinearity. The first principal component (PC1) was introduced into the analysis separately as a control factor [33]. PC1 explained 45.34–78.08% of the total variance for these four categories of factors (Table S3). We used three analysis methods to further reveal the key drivers for microbial CUE along the afforestation chronosequence. Firstly, the partial correlation analysis, linear regression, and PLS-PM analysis were performed to evaluate the total relationship between microbial CUE and potential factors in the afforestation chronosequence [17,31]. Secondly, a Mantel test was performed to determine the A strategy genes associated with microbial CUE. Random Forest was performed to screen the potential functional genes that affect microbial CUE [35]. Thirdly, Pearson correlation analysis was conducted to evaluate the relationships between microbial CUE and potential genes that were screened from the results of the Mantel test and Random Forest. These analyses were conducted using R software v.4.0.3.

## 3. Result and Discussion

**Figure 1:**
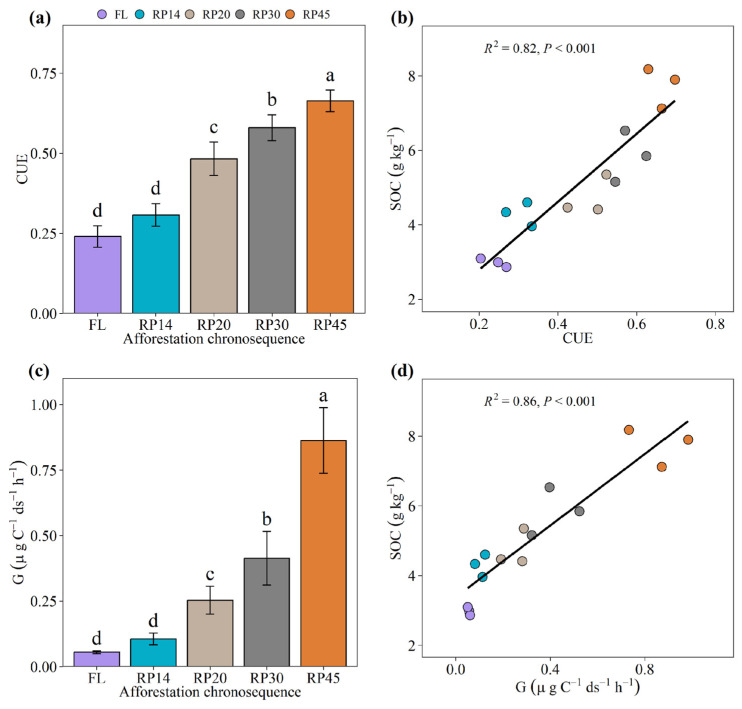
Soil microbial C metabolic properties along the afforestation chronosequence. (**a**) Mean values (±SE, *n* = 3) of microbial carbon use efficiency. (**b**) Relationships of microbial CUE with SOC. The solid lines are regression curves based on linear mixed-effect models, and the shaded areas represent 95% confidence intervals. (**c**) Mean values (±SE, *n* = 3) of soil mass-based growth (G). (**d**) Relationships of growth (G) with SOC. The solid lines are regression curves based on linear mixed-effect models, and the shaded areas represent 95% confidence intervals. Significant differences are denoted by different letters (*p* < 0.05). ds: dry soil; FL: farmland; RP14, RP20, RP30, and RP45 represent that the Robinia pseudoacacia plantations had been restored for approximately 14, 20, 30, and 45 years, respectively. Error bars indicate the standard error of the mean. Different lowercase letters indicate a significant difference (*p* < 0.05) between different age classes, based on a one-way ANOVA followed by an LSD test.

We found that microbial CUE ranged between 0.24 and 0.66 (Figure 1a), consistent with previous empirical and experimental estimates across different land uses and geological contexts [3,36]. The great variation emphasizes the importance of understanding the mechanism of microbial CUE. The increase in microbial CUE after afforestation (*p* < 0.001; Figure 1a) suggests that afforestation promotes microbial communities to invest more in biomass production [17]. The pattern of microbial CUE was similar to that of SOC with afforestation (*R*^2^ = 0.82, *p* < 0.001; Figure 1b), proving that microbial CUE is an important determinant of SOC storage [3,37]. Also, we further found that an increased microbial CUE was largely dependent on the increased microbial growth rather than respiration during the restoration timeline (Figure 1c and Figure S1c). Specifically, the microbial growth rate was greater than the respiration rate after afforestation, which determined the high microbial CUE (Figure S1b,d). The result proves that microbial CUE promotes C accumulation through increased microbial growth [37] and conforms to the soil C pump theory [38]. Therefore, our study provides evidence in the context of afforestation that soil carbon accumulation is largely driven by elevated microbial CUE—a pattern directly influenced by accelerated microbial growth.

**Figure 2:**
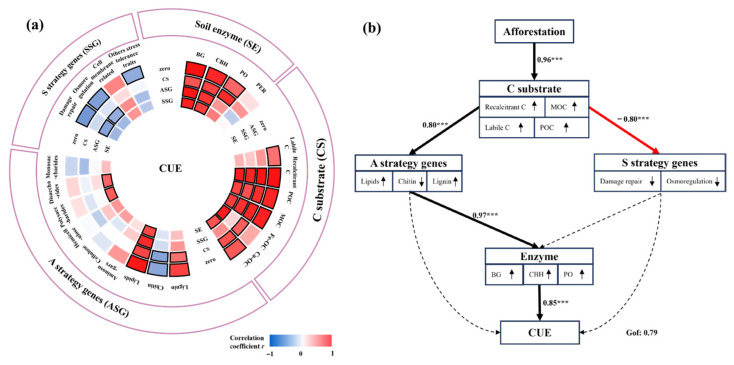
Relationships between microbial C metabolic properties (CUE) and biotic and abiotic factors. (**a**) Partial correlations between soil microbial CUE and four types of factors contributing to soil microbial metabolic properties. The outermost circle shows the factors (i.e., A strategy genes (ASGs), S strategy genes (SSGs), C substrate (CS), and soil enzyme (SE)) whose correlations with microbial CUE are examined. The color of the fan shapes indicates the strength and sign of the correlation, with a black frame line representing the significant correlation at the level of 0.05. Color differences between the zero-order (zero) and controlled factors indicate the level of dependency of the correlation between the microbial CUE and the examined factor on the controlled variable (no change in circle color between the controlled factor and zero-order = no dependency; a decrease/increase in circle color intensity = loss/gain of correlation). (**b**) Directed graph of the partial least squares path model (PLS-PM) showing the effects of C substrates, A strategy genes, S strategy genes, and soil enzymes induced by afforestation on microbial CUE. Single-headed arrows indicate the hypothesized direction of causation. Indicated values are the path coefficients. Black and red arrows indicate positive and negative relationships, respectively. The arrow width is proportional to the strength of the relationship. Models with different structures were assessed using the Goodness of Fit (GOF) statistic, a measure of the overall prediction performance. Asterisks denote levels of significance (*** *p* < 0.001).

The life history strategies of soil microbes determine their metabolic strategies in response to environmental changes and further influence soil C cycling [13,14]. There is a tradeoff between microbial Y, A, and S strategies [39], which determines the direction of microbial metabolism of C [14]. Our results also showed that the Y strategy, characterized by microbial CUE, is correlated with the A and S strategies (Figure 2a). Specifically, the S strategy declined with increasing stand age and exhibited a negative association with microbial CUE (Figure 2a), indicating that elevated microbial CUE results from a reduced investment in stress tolerance [13,40]. Notably, the results of PLS-PM showed that the A strategy explains more variation in the microbial CUE than the S strategy (Figure 2b). Microbial growth is inseparable from resource acquisition in the environment [8,17], and the ability of microorganisms to decompose and utilize soil substrates also determines the metabolic characteristics of microorganisms [16]. Specifically, this reason can be provided as follows: First, afforestation will reduce the stress of microbial survival, thereby reducing the investment in S strategies and shifting to A and Y strategies [14]. An increased Y strategy represents a high microbial yield [13], which has been demonstrated as a key factor in determining high microbial CUE in our study. Second, microbes produce more enzymes to acquire more resources by investing in the A strategy, leading to higher microbial growth [32,41]. Third, microbes have different growth strategies for different substrate accessibilities due to discrepancies in energy investment [11]. Taken together, afforestation-induced increases in microbial CUE can be attributed to the regulation of life history strategies, especially the influence of the A strategy.

**Figure 3:**
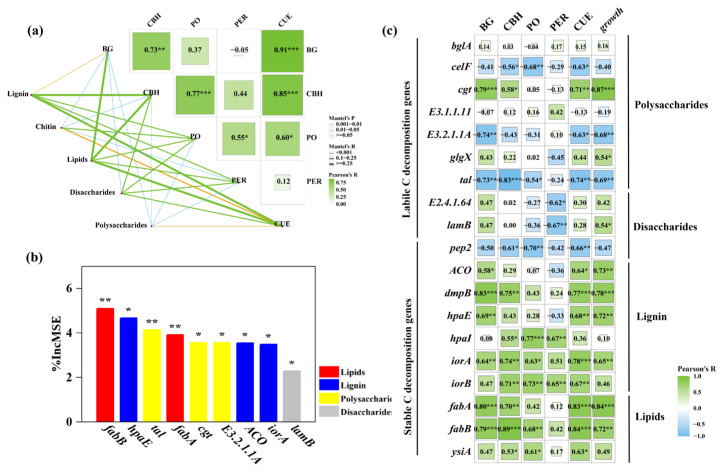
Relationships of microbial C metabolic properties (CUE, growth, and enzyme) and functional genes. (**a**) Relationship between the abundance of the A strategy genes and soil enzyme activity following afforestation (Mantel test and Pearson correlation). (**b**) The first 10 potential functional genes of CUE were selected through Random Forest screening. The accuracy importance measure was computed for each tree and averaged over the forest (5000 trees). The percentage increase in the mean squared error (MSE) of variables was used to estimate the importance of these predictors, and higher MSE% values implied more important predictors. Asterisks denote levels of significance (* *p* < 0.05; ** *p* < 0.01; *** *p* < 0.001). (**c**) Pearson correlations of C-degradation functional genes and C metabolic properties (CUE, respiration, and growth).

We further analyzed functional genes from the A strategy and found that the functional genes involved in the production of enzymes for the decomposition of different C fractions are key factors driving microbial CUE across afforestation (Figure 3a,c). Specifically, we found that the functional genes involved in stable C degradation are more important factors in determining microbial CUE compared with those involved in degrading labile C substrates (Figure 2b and Table S4). In general, the lower C accessibility of stable components reduces the energy investment of microorganisms in respiration to maintain biomass production, causing a lower respiration per unit of biomass growth and thus a higher microbial CUE [10,11]. The results showed that the ability of microorganisms to degrade soil substrates increased, especially for stable organic matter. The functional genes involved in degrading stable C [30,31], such as *fabB*, *hpaE*, *fabA*, *ACO*, and *iorA*, were significantly positively correlated with microbial CUE (Figure 3c, top 5; Table S4). These were the functional genes involved in the production of enzymes for the decomposition of lignin (*hpaE*, *ACO*, and *iorA*) and lipids (*fabB* and *fabA*) [21,31]. Stable C compounds generally have higher nutrient resources and a lower activation energy than labile C compounds, and thus have a greater resource acquisition potential if they can be decomposed by microbes [8,42]. Overall, these results highlight the critical role of functional genes for the A strategy in regulating the microbial CUE along the afforestation chronosequence: forest soils hosting a greater abundance of stable C-degrading genes had stronger resource acquisition capabilities and a higher soil microbial CUE, further supporting that the direct response of soil microorganisms to environmental changes involves changes in life history strategies.

Furthermore, microbial CUE was determined by the C substrate (Figure 2b). In particular, A strategy genes were consistent with changes in C substrates but contrary to those of the S strategy (Figures S2 and S3). Labile C represents a high resource utilization [13]. Therefore, afforestation will promote the A strategy while inhibiting the S strategy by increasing labile C (Table S2). Moreover, afforestation increased the ability of microorganisms to degrade stable C substrates, and this ability was a determinant of microbial CUE (Figure S2). When highly stable C substrates are broken down, they facilitate the more efficient assimilative growth of microorganisms (biomass synthesis) rather than respiration [8]. Consequently, changes in life history strategies induced an increase in microbial CUE through an increased C substrate composition along the afforestation chronosequence. However, the inevitable residue of DNA during DNA extraction could impact the estimation of CUE, necessitating further methodological improvements for more accurate CUE calculations in future research. Additionally, the relatively small sample size from this small watershed may influence the results, and larger-scale spatial sampling would strengthen the assessment of how microbial life history strategies impact CUE along the afforestation chronosequence.

## 4. Conclusions

**Figure 4:**
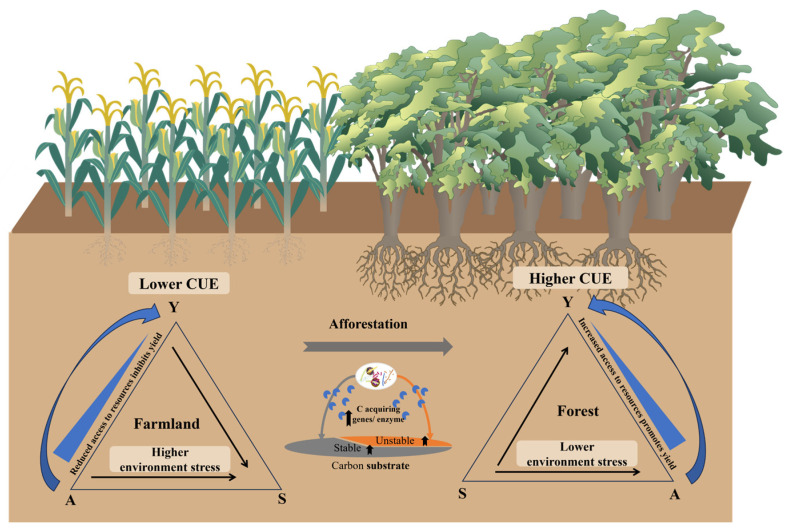
Conceptual diagrams showing regulators and controls of CUE and afforestation effects on CUE by life history strategies.

Our study provides novel evidence through 18O-H^2^O methods that increased microbial CUE promotes SOC accumulation after afforestation. Afforestation regulates microbial CUE by adjusting life history strategies: microbes in the afforestation ecosystem reduce the investment in S strategies and shift to A and Y (microbial CUE) strategies. The A strategy also promotes microbial CUE (Figure 4). Collectively, our study provides novel insights into the tradeoffs of life history strategies (Y–A–S) for microbial CUE in the afforestation ecosystem.

## Data Availability

The original contributions presented in this study are included in the article/Supplementary Materials. Further inquiries can be directed to the corresponding authors.

## Supplementary Materials

The following supporting information can be downloaded at https://www.mdpi.com/article/10.3390/microorganisms13122870/s1. Table S1: Geographical features and plant characteristics from the different study sites. Table S2: Changes in soil and microbial properties variabilities (soil substrates, microbial biomass C, N, and P, and microbial enzymes) along the afforestation chronosequence. Table S3: Results of principal components analysis (PCA) of A strategy genes, S strategy genes, soil enzyme, and C substrate. Table S4: The first 30 potential functional genes of CUE were selected through Random Forest screening. Figure S1: Soil microbial C metabolic properties along the afforestation chronosequence. Figure S2: The abundance of microbial A strategy genes along the afforestation chronosequence. Figure S3: The abundance of microbial S strategy genes along the afforestation chronosequence.

## Abbreviations

The following abbreviations are used in this manuscript:

CUE	N mineralization rates
SOC	Soil organic C
TN	Total N
MAT	Mean annual temperature
MAP	Mean annual precipitation
BD	Soil bulk density

## Appendix A. Method for the Determination of Ca-OC and Ca_exe_

Ca-OC was determined using the Na_2_SO_4_ method. A 2.0000 g soil sample was weighed into a 50 mL centrifuge tube with a lid. Add 20 mL 0.5 mol/L sodium sulfate solution (adjusted to pH 7.0 with sodium hydroxide) into the tube, cover the tube, shake well, and place the sample in a shaker table at 25 °C and 180 r/min for 2 h. After centrifugation at 4000× *g* for 20 min, the supernatant and precipitate were separated. The extraction process was repeated several times until the supernatant no longer contained calcium. Then add 20 mL 10 g/L sodium sulfate solution to the precipitation, shake well and then centrifuge, again add 20 mL 10 g/L sodium sulfate solution to the residue in the centrifuge tube, shake well and then centrifuge, repeatedly use 10 g/L sodium sulfate solution to wash the residue in the centrifuge tube until the cleaning solution was colorless. The residue in the centrifuge tube was freeze-dried. Then the content of calcium-bound organic carbon (Ca-OC) in soil samples was calculated according to the formula:

$$\mathrm{Ca}-\mathrm{OC}=\mathrm{OC}-{\mathrm{OC}}_{{\mathrm{Na}}_{2}{\mathrm{SO}}_{4}}$$

where OC and ${\mathrm{OC}}_{{\mathrm{Na}}_{2}{\mathrm{SO}}_{4}}$ are the organic carbon content of soil and Na_2_SO_4_-treated soil residues, respectively. Exchangeable Ca was extracted with 1 M ammonium acetate (pH 7.0); afterward, the extracted Ca was determined by atomic absorbance.

## Appendix B. Method for the Determination of Fe-OC and Fe_d_

Fe-OC was determined using the citrate–bicarbonate–dithionite (CBD) method. Sodium chloride (NaCl) was used instead of CBD in control experiments. Specifically, two parts of 0.500 g dried soils (<53 μm) were extracted with citrate–bicarbonate (0.27 mol/L trisodium citrate and 0.11mol/L sodium bicarbonate mixed solution, pH 7.3) and NaCl–bicarbonate (1.6 mol/L NaCl and 0.11 mol/L sodium bicarbonate mixed solution, pH 7.3), respectively. The mixtures were preheated in an 80 °C water bath for 15 min. Then, 0.5 g sodium dithionite powder (0.44 g NaCl for the control) was added. The mixture was kept warm for 15 min with constant shaking and then centrifuged at 4000× *g* for 20 min. The soil residue was rinsed five times with 10 mL of deionized water. The final residue was dried and ground. The supernatant was collected and filled with deionized water to 100 mL for determination of Fe_d_. Fe-OC was determined as:

$$\mathrm{Fe}-\mathrm{OC}={\mathrm{OC}}_{\mathrm{NaCl}}-{\mathrm{OC}}_{\mathrm{CBD}}$$

where OC_NaCl_ and OC_CBD_ are the organic carbon content of NaCl- and CBD-treated soil residues, respectively. The OC/Fe molar ratio was used as an indicator of Fe–OM interaction type, which is 1.0 for sorption and >6 for co-precipitation [43].
