# Supplementary material for: Soil Microbial Life History Strategies Drive Microbial Carbon Use Efficiency Following Afforestation
Source: Microorganisms. 2025 Dec 17;13(12):2870. doi: 10.3390/microorganisms13122870 (PMC12735410; doi:10.3390/microorganisms13122870)
Supplement: Supplementary file 1 [file microorganisms-13-02870-s001.zip › microorganisms-3991548-supplementary.pdf]

Supplementary Materials

Soil Microbial Life History Strategies Drive Microbial Carbon Use Efficiency Following Afforestation

Hongyan Cheng [1,2], Haoyuan Chong [3], Minshu Yuan [2], Chengjie Ren [3,] *, Jun Wang [1,4,] * and Fazhu Zhao [1,4]

1 Shaanxi Key Laboratory of Earth Surface System and Environmental Carrying Capacity, College of Urban and Environmental Science, Northwest University, Xi'an 710127, China

2 Yangling Xinhua Ecology Technology Co., Ltd., Yangling 712100, China

3 College of Agronomy, Northwest A&F University, Yangling 712100, China

4 Shaanxi Key Laboratory for Carbon Neutral Technology, Northwest University, Xi'an 710127, China

* Correspondence: rencj1991@nwsuaf.edu.cn (C.R.); wangj@nwu.edu.cn (J.W.)

Table S1. Geographical features and plant characteristics from the different study sites.

Table S2. Changes of soil and microbial properties variabilities (soil substrates, microbial biomass C, N and P and microbial enzyme) along the afforestation chronosequence. Different letters indicate significant differences (ANOVA, *p*< 0.05, Tukey's HSD post-hoc analysis among different the afforestation chronosequence.

Table S3. Results of principal components analysis (PCA) of A strategy genes, S strategy genes, soil enzyme and C substrate.

Table S4. The first 30 potential functional genes of CUE were selected by random forest screening. The accuracy importance measure was computed for each tree and averaged over the forest (5000 trees). Percentage increase in the MSE (mean squared error) of variables was used to estimate the importance of these predictors, and higher MSE% values implied more important predictors. Asterisks denote levels of significance (*p < 0.05; **p < 0.01; ***p < 0.001).

Figure S1. Soil microbial C metabolic properties along the afforestation chronosequence. Mean values (±SE, n = 3) of (a) microbial biomass turnover rate (MTR). (b) biomass- specific growth (G_m), (c) soil mass- based respiration (*R*) and (d) biomass- specific respiration (R_m) under different N levels. Significant differences are denoted by different letters (p < .05). ds, dry soil; C_{mic}, microbial biomass C. FL: farmland; 14yr, 20yr, 30yr, and 45yr represent

that the *Robinia pseudoacacia* plantations had been restored for approximately 14-, 20-, 30-, and 45-year, respectively. Error bars indicate standard error of the mean. Different lowercase letters indicate a significant difference ($P < 0.05$) between different age classes, based on a oneway ANOVA followed by an LSD test.

Figure S2. The abundance of microbial A strategy genes along the afforestation chronosequence.

Figure S3. The abundance of microbial S strategy genes along the afforestation chronosequence.

Table S1. Geographical features and plant characteristics from the different study sites.

Sites[A]	Stand age (years)	Geographical coordinates	Slope gradient (°)	Elevation (m)
FL	0	36°52′05.30″ N 109°20′58.87″E	5	1203
RP14	14	36°51′09.99″ N 109°20′54.68″E	20	1241
RP20	20	36°52′08.48″N 109°21′07.58″E	15	1302
RP30	30	36°51′39.33″ N 109°21′19.75″E	20	1265
RP45	45	36°52′16.78″ N 109°20′53.29″E	25	1291

A FL: farmland; RP14, RP20, RP30, and RP45represent that the *Robinia pseudoacacia* plantations had been restored for approximately 14-, 20-, 30-, and 45-year, respectively.

Table S2. Changes of soil and microbial properties variabilities (soil substrates, microbial biomass C, N and P and microbial enzyme) along the afforestation chronosequence. Different letters indicate significant differences (ANOVA, *p*< 0.05, Tukey's HSD post-hoc analysis among different the afforestation chronosequence.

	**Variables**	**Afforestation chronosequence**						
		**FL**	**RP14**	**RP20**	**RP30**	**RP45**	**F(10,4)**	***P***
**Plant**	**Litter C ($g{\cdot}kg^{-1}$)**	206.46(32.89)c	352.61(11.69)b	347.27(18.07)b	381.37(21.96)ab	425.59(8.12)a	16.155	<0.01
**Soil properties**	**SM(%)**	12.48 (0.14)ab	13.58 (0.64)a	12.1 (0.33)b	11.94 (0.46)b	12.75 (0.42)ab	2.271	0.134
	**pH**	8.28 (0.04)c	8.39 (0.03)ab	8.3 (0.03)bc	8.45 (0.02)a	8.3 (0.03)bc	6.345	<0.01
	**BD (g/cm^{-3})**	1.28 (0.03)a	1.21 (0.04)a	1.1 (0.11)a	1.16 (0.05)a	1.06 (0.06)a	1.777	0.21
	**SOC (g/kg)**	2.98 (0.07)d	4.3 (0.19)c	4.74 (0.31)c	5.84 (0.4)b	7.73 (0.32)a	40.978	<0.001
	**TN (g/kg)**	0.26 (0.03)b	0.42 (0.06)a	0.53 (0.06)a	0.51 (0.04)a	0.44 (0.01)a	6.904	<0.01

	**TP (g/kg)**	0.57 (0.01)c	0.6 (0)a	0.58 (0)b	0.57 (0)cd	0.56 (0)d	15.571	<0.001
	**NH^{4+} (mg/kg)**	1.73 (0.08)b	2.14 (0.1)b	3.45 (0.15)a	3.6 (0.21)a	1.95 (0.02)b	47.799	<0.001
	**NO^{3-} (mg/kg)**	3.55 (0.51)bc	4.42 (0.79)b	1.5 (0.43)bc	1.24 (0.51)c	13.06 (1.75)a	26.604	<0.001
	**AP (mg/kg)**	2.4 (0.4)c	4.19 (0.13)b	5.73 (0.28)a	4.95 (0.29)ab	5.16 (0.17)a	22.487	<0.001
	**SOC : TN (C:N)**	11.86(1.26)b	10.46(0.94)b	9.06(0.83)b	11.66(1.19)b	17.46(1.00)a	9.194	<0.01
	**SOC : TP (C:P)**	5.22(0.09)d	7.22(0.29)c	8.16(0.48)c	10.28(0.69)b	13.86(0.48)a	52.863	<0.001
	**TN : TP N:P)**	0.45(0.05)b	0.71(0.09)a	0.91(0.09)a	0.89(0.07)a	0.80(0.02)a	7.368	<0.01
**C accessibility**	**MOC($g \cdot kg^{-1}$)**	2.43(0.07)d	3.69(0.06)c	3.87(0.19)c	4.67(0.18)b	6.46(0.17)a	100.733	<0.001
	**POC($g \cdot kg^{-1}$)**	0.56(0.07)b	0.61(0.06)b	0.88(0.19)ab	1.17(0.18)a	1.28(0.17)a	4.776	<0.05

	**Ca-OC($g\cdot kg^{-1}$)**	1.64(0.16)d	2.67(0.16)bc	3.27(0.41)b	2.12(0.19)cd	6.32(0.2)a	57.667	<0.001
	**Fe-OC($g\cdot kg^{-1}$)**	1.39(0.01)b	0.2(0.01)c	0.15(0.00)c	0.39(0.03)c	2.59(0.35)a	43.302	<0.001
	**Labile C ($g\cdot kg^{-1}$)**	0.77(0.2)b	1.19(0.19)ab	1.26(0.16)ab	1.43(0.52)ab	1.82(0.23)a	1.73	0.22
	**Recalcitrant C ($g\cdot kg^{-1}$)**	2.21(0.13)d	3.11(0.02)c	3.48(0.15)c	4.41(0.28)b	5.91(0.09)a	78.819	<0.001
	**POC (%)**	18.64(2.36)a	14.06(1.01)a	18.1(2.78)a	19.9(2.62)a	16.42(1.86)a	1.034	0.436
	**MOC (%)**	81.41(2.85)a	86.28(4.76)a	82.64(8.81)a	81.05(8.28)a	83.96(5.44)a	0.109	0.976
	**Ca-OC (%)**	54.92(5.56)b	62.17(2.78)b	67.71(10.09)ab	35.94(3.15)c	81.83(2.02)a	9.266	<0.01
	**Fe-OC (%)**	46.77(1.42)a	4.69(0.07)c	1.22(1.06)c	6.62(0.02)c	33.54(4.55)b	86.946	<0.001
**Mineral protection**	**Fe_d (g/kg)**	0.16(0.02)ab	0.19(0.01)a	0.14(0.02)ab	0.12(0.01)b	0.16(0.01)ab	2.955	0.075
	**Ca_{exe} (g/kg)**	7.37(0.47)ab	9.44(0.76)a	8.42(0.56)a	5.1(0.79)b	8.27(1.69)a	2.924	0.077

	**OC/Fe molar ratio**	43.71(5.95)b	4.93(0.05)c	4.34(0.25)c	14.83(0.17)c	76.49(6.97)a	57.336	<0.001
**Microorganism**	**MBC（mg kg $^{-1}$)**	60.27 (4.62)d	99.26 (8.82)d	240.53 (31.67)c	367.73 (33.41)b	521.54 (8.9)a	79.493	<0.001
	**MBN（mg kg $^{-1}$)**	7.39 (1.36)c	16.24 (1.85)c	33.13 (4.33)b	46.43 (7.91)b	68 (3.65)a	29.219	<0.001
	**MBP（mg kg $^{-1}$)**	5.55 (0.24)d	6.1 (0.3)d	9.7 (0.22)b	8.36 (0.19)c	13.09(0.44)a	109.103	<0.001
	**BG (nmol g $^{-1}$ h $^{-1}$)**	30.02 (2.92)d	46.42 (1.5)c	54.86 (2.8)c	94.22 (3.71)b	124.8(7.71)a	81.366	<0.001
	**CBH (nmol g $^{-1}$ h $^{-1}$)**	5.06(0.24)c	7.8(0.73)b	10.5(1.48)a	10.92(0.49)a	11.01(0.46)a	10.371	<0.01

**PO (nmol g $^{-1}$ h $^{-1}$)**	1.87(0.04)c	2.24(0.16)b	3.15(0.13)a	3.15(0.06)a	2.45(0.1)b	26.898	<0.001
**PER (nmol g $^{-1}$ h $^{-1}$)**	2.12(0.03)d	3.06(0.1)a	3.06(0.03)a	2.75(0.03)b	2.44(0.06)c	50.037	<0.001

soil organic carbon (SOC), total nitrogen (TN), total phosphorus (TP), microbial biomass carbon (MBC), microbial biomass nitrogen (MBN), available phosphorus (AP)microbial biomass phosphorus (MBP); β-1,4-glucosidase (BG, EC 3.2.1.21), β-1,4-N-acetylglucosaminidase (NAG, EC 3.1.6.1), leucine aminopeptidase, (LAP, EC 3.4.11.1), alkaline phosphatase (ALP, EC 3.1.3.2).

Table S3. Results of principal components analysis (PCA) of A strategy genes, S strategy genes, soil enzyme and C substrate.

	Factors	Loading
	**Monosaccharides**	0.418
	**Disaccharides**	0.32
	**Polysaccharides**	0.349
	**Hemicellulose**	0.185
**A strategy genes**	**Cellulose**	0.372
	**Aminosugars**	-0.101
	**Lipids**	-0.323
	**Chitin**	0.396
	**Lignin**	-0.397
	**Cumulative (%)**	45.34
	**Damage repair**	0.555
	**Osmo regulation**	0.748
**S strategy genes**	**Cell membrane related**	-0.275
	**Others stress tolerance traits**	0.339
	**Cumulative (%)**	78.08
**Soil**	**BG**	0.426

**enzyme**	**CBH**	0.608
	**PO**	0.558
	**PER**	0.371
	**Cumulative (%)**	62.17
	**Labile C**	0.385
	**Recalcit ant C**	0.442
	**POC**	0.403
**C fraction**	**Ca-OC**	0.448
	**MOC**	0.461
	**Fe-OC**	0.284
	**Cumulative (%)**	72.84

Table S4. The first 30 potential A strategy genes of CUE were selected by random forest screening. The accuracy importance measure was computed for each tree and averaged over the forest (5000 trees). Percentage increase in the MSE (mean squared error) of variables was used to estimate the importance of these predictors, and higher MSE% values implied more important predictors. Asterisks denote levels of significance (*p < 0.05; **p < 0.01; ***p < 0.001).

	%IncMSE	%IncMSE.pval	IncNodePurity	IncNodePurity.pval	%IncMSE.sig
**fabB**	5.092649	0.009901	0.037191	0.009901	**
**hpaE**	4.67542	0.039604	0.014044	0.108911	*
**tal**	4.143349	0.009901	0.027938	0.009901	**
**fabA**	3.916346	0.009901	0.028865	0.009901	**
**cgt**	3.558295	0.019802	0.021842	0.039604	*
**E3.2.1.1A**	3.554125	0.029703	0.01878	0.029703	*
**ACO**	3.551282	0.019802	0.006195	0.336634	*
**iorA**	3.492099	0.029703	0.019941	0.029703	*
**ysiA**	2.692054	0.069307	0.013746	0.09901	
**celF**	2.505194	0.059406	0.014919	0.108911	
**lamB**	2.288835	0.039604	0.001859	0.871287	*
**hpaI**	2.09307	0.079208	0.00481	0.366337	

**iorB**	1.99917	0.089109	0.012224	0.089109
**glgX**	1.348581	0.09901	0.003532	0.524752
**malL**	1.323654	0.09901	0.003035	0.633663
**E2.4.1.64**	1.220365	0.118812	0.002573	0.633663
**E3.1.1.11**	1.211275	0.19802	0.000177	1
**bglA**	0.885935	0.138614	0.002949	0.693069
**pep2**	0.783117	0.227723	0.010082	0.148515
**dmpB**	0.776207	0.138614	0.005885	0.207921
**hpaH**	0.671797	0.178218	0.011285	0.148515
**treS**	0.624001	0.178218	0.000959	0.950495
**ptsG**	0.57694	0.237624	0.01132	0.158416
**dmpD**	0.542367	0.217822	0.009251	0.237624
**ligB**	0.540699	0.188119	0.002641	0.653465
**E3.2.1.58**	0.426503	0.287129	0.005856	0.346535
**glgA**	0.392466	0.247525	0.004485	0.49505
**treZ**	0.367526	0.227723	0.001929	0.910891
**bglX**	0.281729	0.257426	0.000851	0.980198
**ligA**	0.218054	0.277228	0.000805	0.990099
**nagD**	0.205811	0.386139	0.001418	0.881188
**pgmB**	0.170786	0.376238	0.001666	0.930693
**amyA**	0.148114	0.326733	0.000376	1

**rfbF**	0.143454	0.207921	0.000427	0.990099
**TREH**	0.133795	0.306931	0.00074	0.970297
**E2.4.1.7**	-0.08145	0.336634	0.003036	0.732673
**hpaG**	-0.11153	0.316832	0.000349	1
**hpaF**	-0.17549	0.287129	0.000465	0.990099
**glgM**	-0.65038	0.544554	0.00107	0.930693
**E3.2.1.3**	-0.84102	0.524752	0.007281	0.277228
**mhpA**	-0.95074	0.623762	0.002766	0.673267
**hpaC**	-0.97578	0.554455	0.000721	0.970297
**bglB**	-1.00706	0.584158	0.010422	0.138614
**praC**	-1.05955	0.60396	0.000288	1
**glgP**	-1.07829	0.524752	0.002571	0.70297
**E2.4.1.4**	-1.3601	0.70297	0.002119	0.742574
**NAGLU**	-1.42962	0.792079	0.004228	0.613861
**catA**	-1.46132	0.742574	0.002973	0.732673
**CBH2**	-1.59284	0.712871	0.004533	0.475248
**malQ**	-1.60568	0.742574	0.001087	0.990099
**TC.GPH**	-1.67038	0.861386	0.00505	0.386139
**maa**	-1.71026	0.80198	0.001073	0.960396
**K07485**	-1.95942	0.891089	0.000565	0.990099
**hpaD**	-2.17157	0.910891	0.002318	0.881188

**lacA**	-2.24154	0.980198	0.001936	0.871287

Figure.S1.

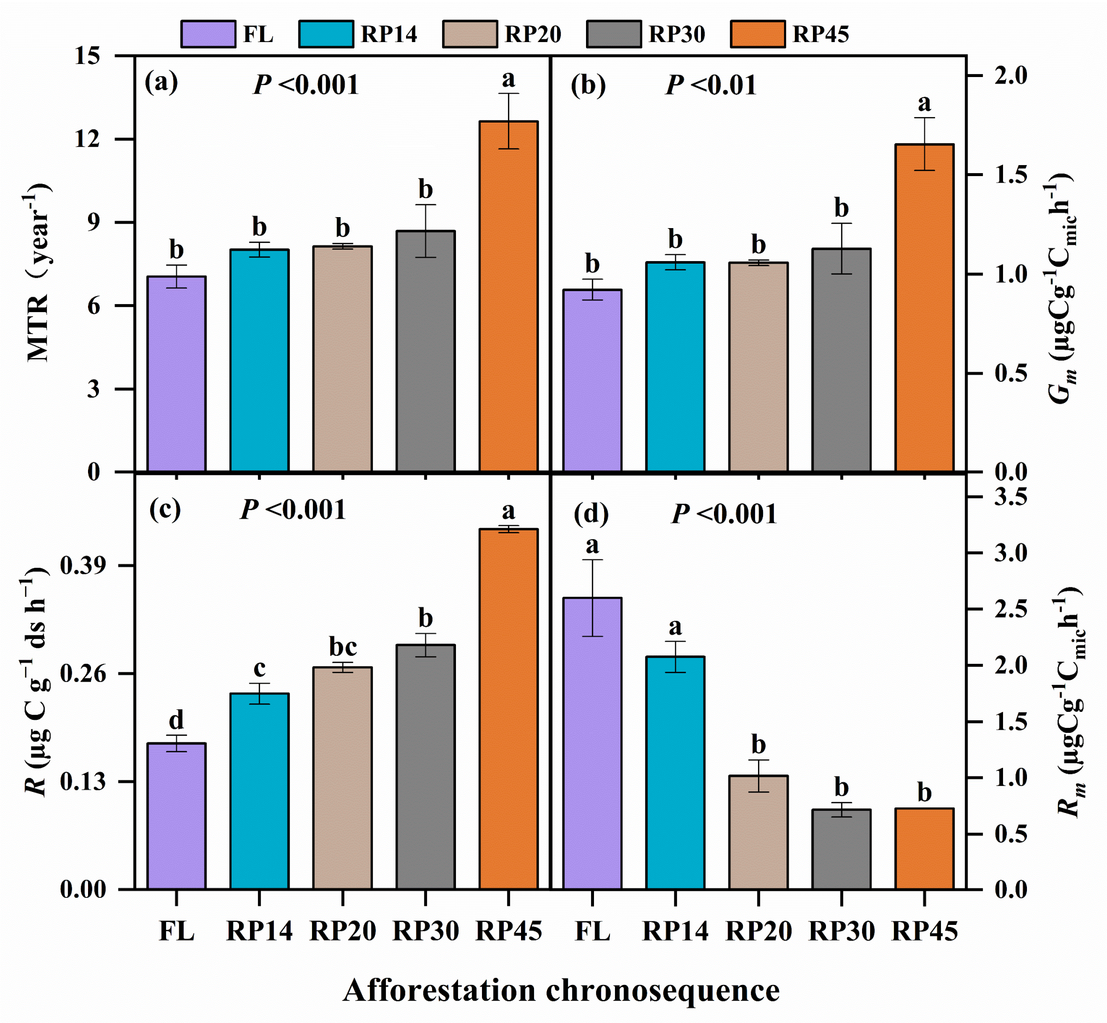

Figure S1. Soil microbial C metabolic properties along the afforestation chronosequence. Mean values (±SE, n = 3) of (a) microbial biomass turnover rate (MTR). (b) biomass- specific growth (G_m), (c) soil mass- based respiration (*R*) and (d) biomass- specific respiration (R_m) under different N levels. Significant differences are denoted by different letters ($p < .05$). ds, dry soil; C_{mic}, microbial biomass C. FL: farmland; 14yr, 20yr, 30yr, and 45yr represent that the *Robinia pseudoacacia* plantations had been restored for approximately 14-, 20-, 30-, and 45-year, respectively. Error bars indicate standard error of

the mean. Different lowercase letters indicate a significant difference ($P < 0.05$) between different age classes, based on a oneway ANOVA followed by an LSD test.

Figure S2.

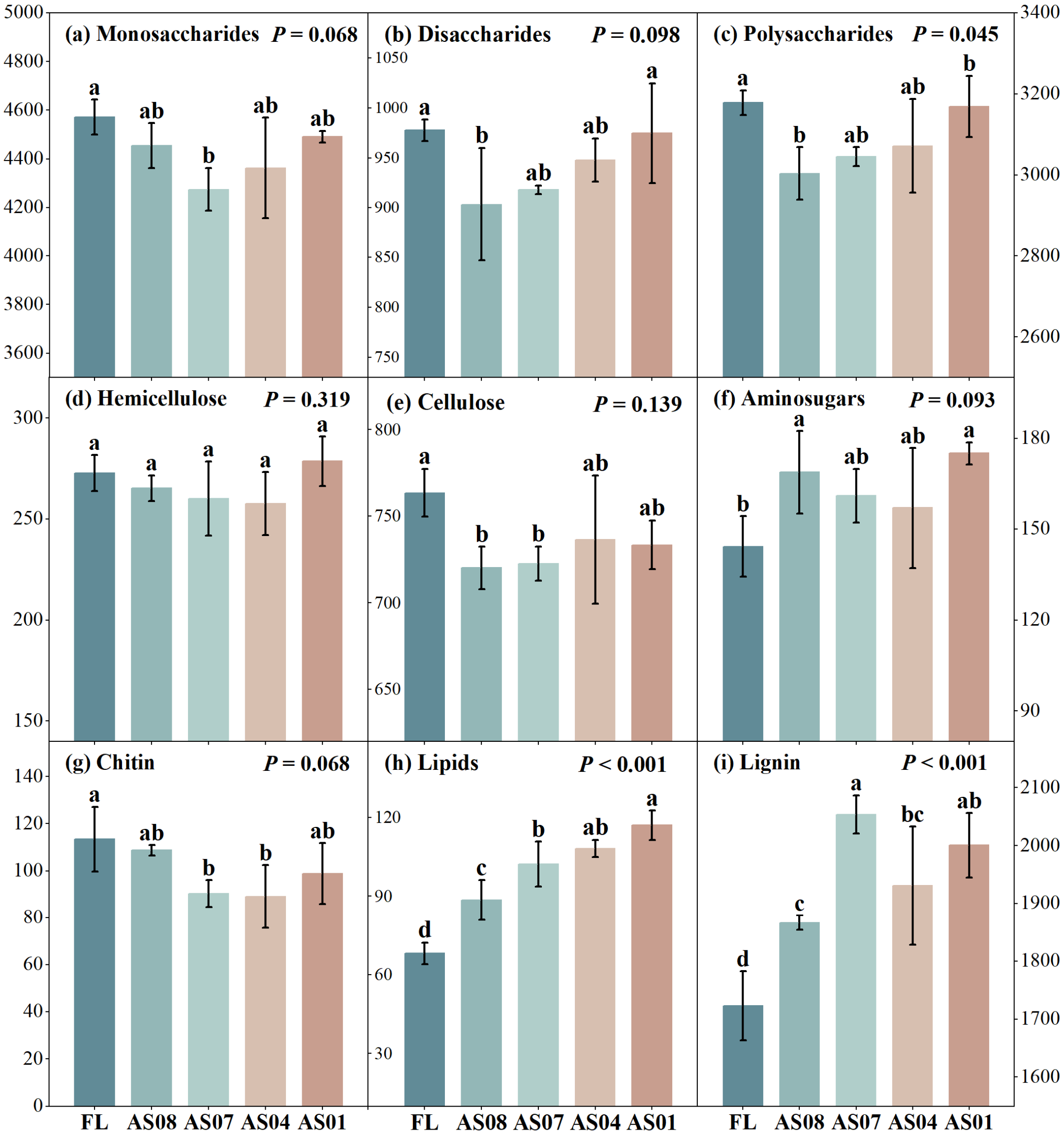

Figure S2. The abundance of microbial A strategy genes along the afforestation chronosequence.

Figure S3.

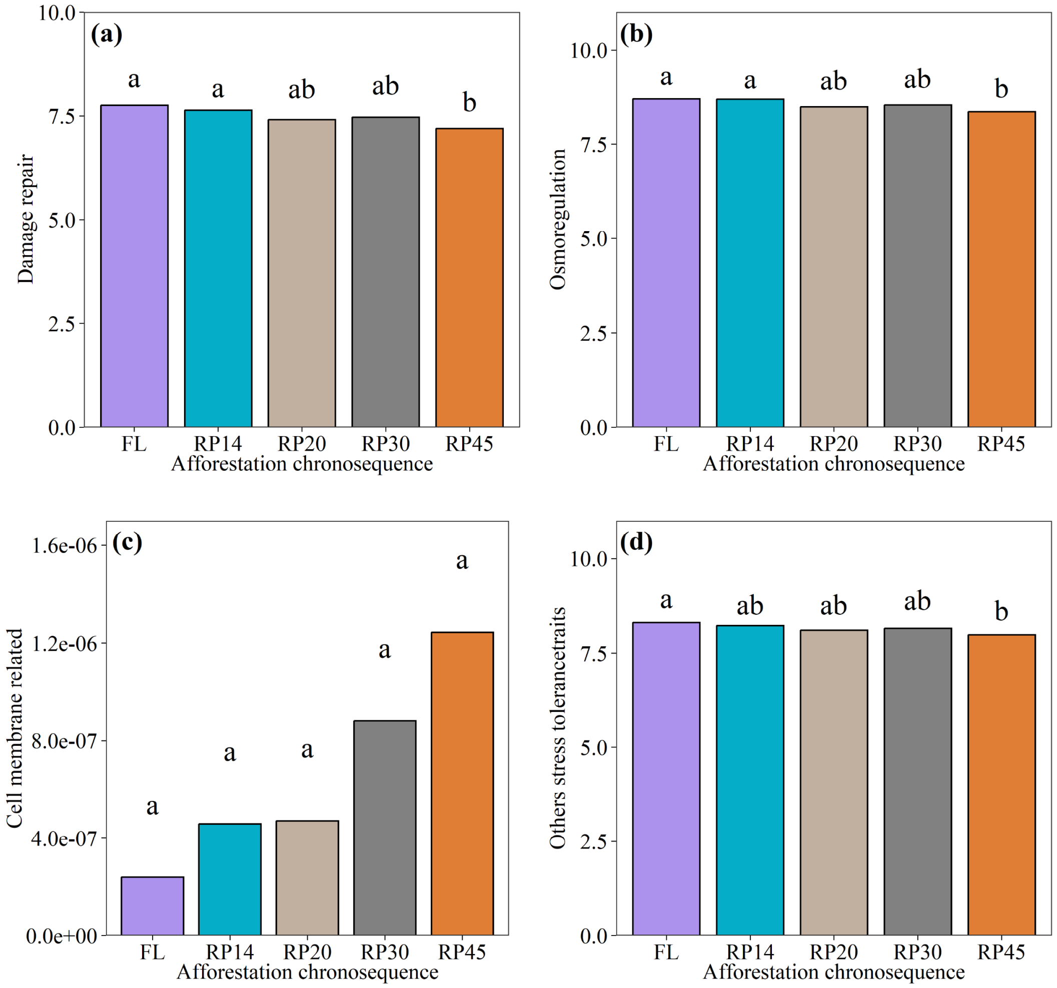

Figure S3. The abundance of microbial S strategy genes along the afforestation chronosequence.
